# Power-Intent Systolic Array Using Modified Parallel Multiplier for Machine Learning Acceleration

**DOI:** 10.3390/s23094297

**Published:** 2023-04-26

**Authors:** Kashif Inayat, Fahad Bin Muslim, Javed Iqbal, Syed Agha Hassnain Mohsan, Hend Khalid Alkahtani, Samih M. Mostafa

**Affiliations:** 1 Department of Electronics Engineering, Incheon National University, Incheon 22012, Republic of Korea; 2 Faculty of Computer Sciences and Engineering, GIK Institute of Engineering Sciences and Technology, Topi 23460, Pakistan; 3 Department of Computer Systems Engineering, University of Engineering and Applied Sciences, Swat 19201, Pakistan; 4 Optical Communication Laboratory, Ocean College, Zhejiang University, Zheda Road 1, Zhoushan 316021, China; 5 Department of Information Systems, College of Computer and Information Sciences, Princess Nourah bint Abdulrahman University, P.O. Box 84428, Riyadh 11671, Saudi Arabia; 6 Computer Science Department, Faculty of Computers and Information, South Valley University, Qena 83523, Egypt

**Keywords:** machine learning, deep learning, accelerators, power-intent, systolic arrays

## Abstract

Systolic arrays are an integral part of many modern machine learning (ML) accelerators due to their efficiency in performing matrix multiplication that is a key primitive in modern ML models. Current state-of-the-art in systolic array-based accelerators mainly target area and delay optimizations with power optimization being considered as a secondary target. Very few accelerator designs directly target power optimizations and that too using very complex algorithmic modifications that in turn result in a compromise in the area or delay performance. We present a novel Power-Intent Systolic Array (PI-SA) that is based on the fine-grained power gating of the multiplication and accumulation (MAC) block multiplier inside the processing element of the systolic array, which reduces the design power consumption quite significantly, but with an additional delay cost. To offset the delay cost, we introduce a modified decomposition multiplier to obtain smaller reduction tree and to further improve area and delay, we also replace the carry propagation adder with a carry save adder inside each sub-multiplier. Comparison of the proposed design with the baseline Gemmini naive systolic array design and its variant, i.e., a conventional systolic array design, exhibits a delay reduction of up to 6%, an area improvement of up to 32% and a power reduction of up to 57% for varying accumulator bit-widths.

## 1. Introduction

Deep learning (DL) has swiftly emerged as an important subclass of machine learning (ML) offering break-neck speeds that are important for a variety of applications in diverse domains such as pattern recognition, image classification, computer vision, etc. [1]. In order to reap the benefits of the deep learning models, tremendous costs need to be incurred though, owing to the requirements of such models to perform several complex and memory-access intensive operations [2]. To accelerate the execution of such operations, deep neural networks (DNNs) rely on dedicated hardware accelerators that can speed-up the most critical computations while being much more energy-efficient compared to the host processors [3]. Such networks are usually characterized by a plethora of multiply and add operations, and thus general matrix multiplication (GEMM) becomes an obvious choice to be accelerated in order to achieve the enormous processing abilities required for ML applications.

Systolic arrays (SAs), since originating in the 1980s, have found a renewed interest due to their crucial role in matrix multiplication acceleration. This is evident from some recent developments regarding DNN acceleration involving big market players such as Google, Nvidia, Samsung, etc. [4,5,6]. A systolic array is a 2D arrangement of processing elements (PEs) arranged in a grid enabling the efficient execution of conventional algorithms, e.g., GEMM, performing single instruction multiple data (SIMD) types of operations [7]. They have been readily adopted in a variety of ML accelerators based upon GEMM due to their properties such as regular structure, reconfigurability, scalability, etc. [8].

The ability of ML accelerators to offer execution efficiency comes at the cost of their requirement of tremendous computing power, which necessitates them to be power efficient as well. While power efficiency is critical to both the cloud and edge aspects of modern artificial intelligence (AI) accelerators, its significance for the edge devices, i.e., within the sensors, cannot be overstated [9]. Moreover, SAs, besides being used in ML accelerators at the cloud, have also found utility in several commercial edge devices (particularly owing to their regularity and enhanced data reuse causing reduced external memory accesses), e.g., the Tesla’s full self-driving chip (FSD) [10] and Google’s edge TPU [11] and have been actively researched to that effect as found in [12,13]. The power aspect of systolic array-based accelerators has been investigated in a variety of recent research articles, e.g., [14,15,16], but the focus in all these works was to use various complex code optimizations to, e.g., either exploit the underlying embedded system hardware or to reduce memory-accesses. Additionally, sparse matrices are frequently encountered in a variety of ML applications, e.g., in recommender systems utilizing product usage data within a catalog, in natural language processing working with text documents and in computer vision involving image processing with monotonic images. Such cases result in the inefficient utilization of SAs because of the PEs having to do multiplication and accumulation (MAC) operations on zero-valued entries of the matrices that are not contributing to the ultimate result, thus resulting in wasted power due to idle PEs [17,18].

This work presents an approach to achieve power efficiency by exploiting fine-grained power gating (particularly for applications involving idle PEs) via the industry standard power-intent specification using a unified power format (UPF) while also utilizing logic-level design optimizations made to the systolic arrays through an open-source systolic array presented in [19] as the baseline. Since the extent of computations performed by MAC operations of the PEs considering ML accelerators is well above 99%, it makes sense to target the MAC block inside the PE for various optimizations [20]. UPF enables power-intent specifications distinctly as compared to the logic-level description of a digital design, thus enabling convenient power optimization by offering effective portability of power-intent description for a wide scope of commercial products throughout the whole cycle of the electronic system design [21]. The logic-level optimizations involve decomposing the larger PE multiplier into multiple smaller sub-multipliers in addition to the multiplier carry propagation adders (CPAs) being replaced by carry save adders (CSAs) in each sub-multiplier as well as the PE.

The major contributions of this paper can be summarized as follows:

- We apply power gating to the conventional systolic array (C-SA) Gemmini accelerator and observe its impact on the various performance parameters by using naive systolic array (N-SA) accelerator as the baseline.
- Since the design mainly suffers from delay degradation due to the low power cells inserted and larger multiplier being used, we then propose a novel micro-architecture termed Power-Intent Systolic Array (PI-SA) utilizing a modified decomposed multiplier in the PEs with the sub-multiplier CPAs replaced by CSAs and power gating the PE multiplier that significantly improves the delay parameter in addition to giving a better mix of the other performance parameters in comparison to both the N-SA and the C-SA designs.
- We perform an initial analysis for a 32-bit-wide accumulator, which is then followed by 16-bit- and 64-bit-wide designs, thus demonstrating the impact of precision scaling on the overall performance parameters.

The rest of the paper is organized as follows. The background information about the various technical aspects making the basis for this research work is presented in Section 2. We present some relevant literature while also describing critical assessment of the published literature in light of our main theme in Section 3. The proposed design is presented in detail in Section 4. Our evaluation methodology and the results are presented in Section 5. Finally, the work is concluded in Section 6.

## 2. Background

### 2.1. Conventional Multipliers in Systolic Arrays

**Figure 1:**
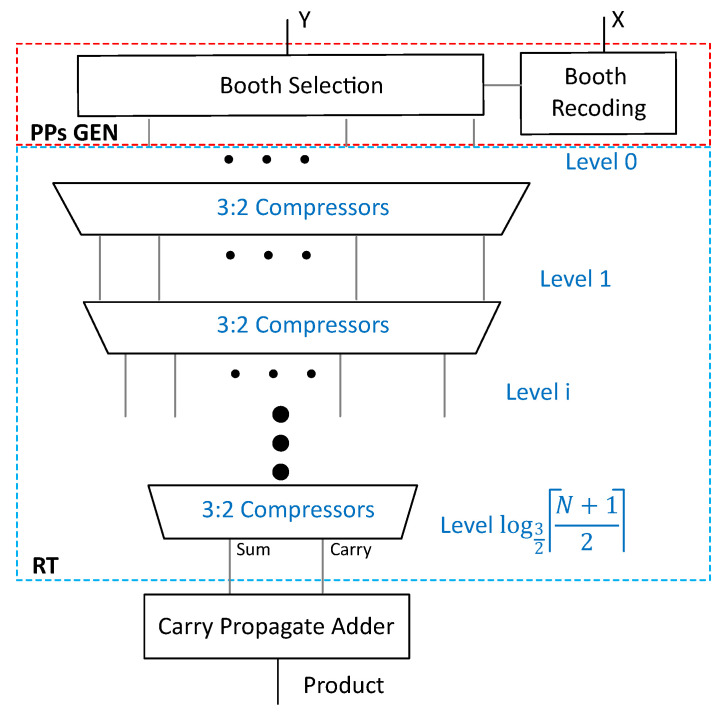
A conventional Radix-4 multiplier overview.

In systolic arrays, multipliers dominate the datapath. Parallel multipliers are composed of three major elements: (1) partial products generation (PPs GEN); (2) partial product reduction trees (RT) (e.g., Wallace tree); and (3) addition of the final sum and carry rows of partial products using a CPA to obtain the final product, as shown in Figure 1.

Consider an M×N-bit multiplier, where $X=x_{M-1}x_{M-2}\cdots x_{1}x_{0}$ and $Y=y_{N-1}y_{N-2}\cdots$$y_{1}y_{0}$ are the multiplier and multiplicand, respectively. The *N* rows of the *M*-bit partial products PP${}_{i,j}$ can be expressed as:

(1) $$\begin{array}{c} PP_{i,j}\;=\;x_{i}y_{j},\;\;\forall i,j,\;0\;\leq \;i\;<\;M,\;0\;\leq \;i\;<\;N, \end{array}$$

Fast multipliers employ the modified Booth encoding algorithm to reduce the height of the partial products [22,23]. By using the radix-*R* = $2^{r}$, *r* > 0, booth multiplier, partial products are reduced from *N* to $\left\lceil \frac{\left(N+1\right)}{r}\right\rceil$, thus reducing the size and speed of the reduction tree. In this paper, we used two radix-4 multipliers: 8-bit×8-bit and 4-bit×4-bit, with $\lceil \frac{\left(8+1\right)}{2}\rceil =5$ and $\lceil \frac{\left(4+1\right)}{2}\rceil =3$ partial products, respectively, and we adopted these structure-level designs from [24].

### 2.2. Naive Systolic Array Architecture

Machine learning accelerators are designed to minimize the computation time; thus, high compute throughput is a necessary requirement for such hardware. Therefore, to efficiently perform GEMMs in machine learning workloads using a large number of PEs configured in two dimensions while avoiding redundant memory accesses, systolic arrays have been employed in many application specific hardwares. Gemmini represents one of the many recent examples that is basically a configurable open source (https://github.com/ucb-bar/gemmini (accessed on 13 April 2023) systolic array accelerator generator (Gemmini RTL generation method explained with details in [25]) introduced by Hasan et al. in [19] that supports multiple dataflows. While the input stationary (IS) dataflow indicates that the input activation matrix is burst-filled, the weight stationary (WS) and the output stationary (OS) indicates the same for the weight and output activation matrices, respectively. Among these dataflows, only the WS and OS dataflow support is provided in Gemmini. The OS dataflow is advantageous from the precision and higher reusability perspective due to the significantly wider bit-width output matrices as compared to the input and the weight matrices.

**Figure 2:**
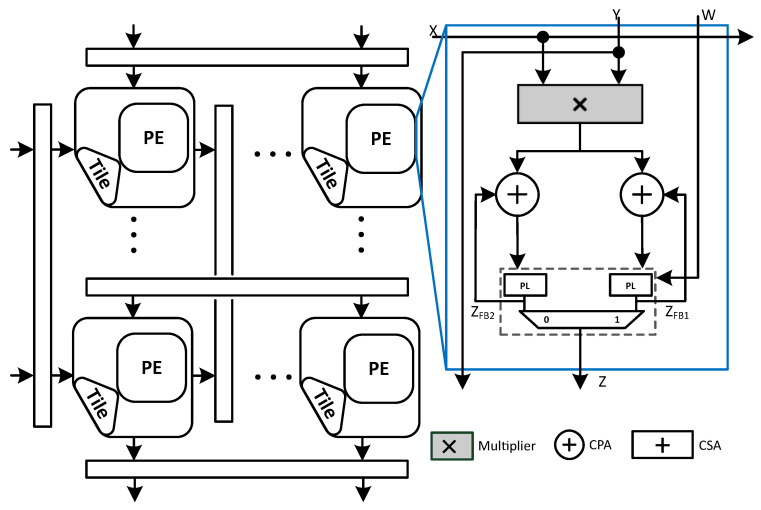
An overview of the Naive systolic array.

Figure 2 shows a Gemmini accelerator with the OS dataflow. This architecture contains a set of PEs arranged in the form of a 2D setting. Each PE contains a multiplier and an adder to perform the MAC computation on its inputs, then passes the computed output and forwards the input values to one or several of the neighboring PEs via pipeline registers in the form of a wave-front flow. Gemmini MAC contains a multiplier and an adder arranged in a cascaded manner with one CPA for multiplier post-processing addition and a separate one for accumulation, so technically it incurs the cost of two CPAs, that is why we call this design as Naive Systolic Array (N-SA).

The input matrix X rows are depicted on the left side in Figure 2, while the columns constituting the input matrices Y and W are shown at the top of the systolic array to accomplish the GEMM operation as mathematically shown by (2):

(2) Z=X×Y+W

where X and Y represent the matrices serving as inputs for the GEMM computation, Z represents the resultant matrix while the W indicates the bias matrix, i.e., the accumulator preload considering the OS type of dataflow. The Gemmini accelerator accomplishes the MAC computation utilizing mixed precision. That is why the MAC consists of a small bit-width (e.g., 4-bit, 8-bit) signed multiplier and a large bit-width (e.g., 32-bit, 64-bit) adder (CPA) to perform multiplication and accumulation, respectively. To maintain a higher accuracy in machine learning applications, usually, the accumulation is performed with a bit-width that is higher than the input bit-width (e.g., 32 bits).

Furthermore, N-SA based Gemmini accelerators have two additional components in each processing element: double buffers (for simplicity, we omitted it from the rest of the figures) (enclosed in dashed lines) for full utilization of MACs, and peripheral logic (PL) to handle rounding of the final output. The double buffer is divided into two registers so that the input loading and the previous result propagation can be carried out at the same time while the current compute cycle is running. This can be graphically seen in the Figure 2.

### 2.3. Power Optimization

Power consumption in ML accelerators is of primary importance and it is necessary to take steps to optimize it. We do so by utilizing power gating that effectively turns off a block (a module or a group of cells) that is not in active use at a particular instance of time. In particular, we perform state-retention power gating that ensures the saving of some system states in the power-switchable block. This is accomplished by special registers called the state-retention registers.

Power-intent description basically divides the overall system into multiple distinct blocks called power domains (PDs) with each domain possibly consisting of several module instances and having its own nominal conditions. This division of the design space into domains enables some parts of the circuit to be switched-off on demand with convenience. In addition to the retention registers, isolation cells normally placed at the output of the switchable domain ensure that the always-on domains always keep on receiving valid logic signals from the switchable power domains [26]. The ability to switch off the main supply to the switchable domain is accomplished by power switches that are inserted during the physical implementation of the design.

A power management unit (PMU) is necessary to be instantiated in the always-on domain to provide the various signals necessary to accomplish power gating in an appropriate manner. The simplified algorithmic description of the PMU with the “isolation enable” and “retention enable” output signals pertinent for logic synthesis and power-shutoff (PSO) enable input signal pso_enable is given in Algorithm 1.
**Algorithm 1:** Power Management Unit Algorithm**Input**: Power gating enable signal pso_enable;**Output**: Enable signals for low power logic insertion;**if** *(pso_enable == 1)* **then**( retention enabled; isolation enabled;**else**( retention disabled; isolation disabled;**end**(

## 3. Related Work

Our work is mainly related to making micro-architectural changes to the systolic array to enable power-intent description, thus obtaining power optimized ML acceleration. The overall performance enhancement is thereafter achieved by decomposing the MAC multiplier into multiple sub-multipliers and making further modifications to these resulting sub-multipliers. We have thus categorized our literature review into three parts and then we critically analyze them in unison.

### 3.1. Low Power Accelerators

Various research activities have been found in the literature that are targeting power-efficient hardware accelerators for ML applications while focusing on different options to achieve power optimization. Ref. [16] includes a comprehensive survey of various optimizations performed at the hardware architecture level to optimize the power consumption of DNNs. The authors present an overview of various architectures based on CPUs, GPUs, ASICs and FPGAs used for DNN acceleration and then present an overview of distinct work performed in optimizing each class of architecture. The work, however, only focuses on memory-access optimizations when considering ASIC-based accelerators.

Similarly, the authors in [14] identify two aspects of ML accelerators that can be targeted for making them energy-efficient. These include factoring out various elements from the accelerator design and catering to inefficient memory transfers in such accelerators. The authors in this work target the latter while targeting a 65 nm process and comparing its performance with accelerators based on a multicore CPU and GPU. This shall, however, obviously include various complicated coding optimizations, e.g., by hard-coding activities such as loop tiling and memory partitioning to achieve the full capacity of these micro-architectural modifications.

The authors in [27] utilize approximate computing via quantization in Convolutional Neural Networks (CNNs) to reduce the bit requirement of encoding the weights and inputs at each layer. This results in a loss of accuracy, but leads to energy efficiency when performing the implementation at 40nm technology while considering various CNN architectures for image classification. The same authors in [28] utilize another approximate computing technique for CNNs termed as dynamic-voltage-accuracy-frequency-scaling (DVAFS) that dynamically scales the voltage and frequency based on accuracy requirements to considerably reduce the dynamic power consumption of the network. The results are supported by measurements made using Envision [29], i.e., a CNN processor with 28nm technology implementation armed with the proposed DVAFS technique. The power efficiency in [29] is again improved by dynamically varying the threshold voltage and hence the supply voltage to cater to varying precision requirements.

### 3.2. Re-Configurable Accelerators

In literature, different re-configurable MAC architectures are available for ML accelerators that are usually designed following either parallel or serial approaches [30,31,32,33] through bit-decomposition technique. Bit Fusion [30] is one of the recent parallel re-configurable accelerators which can execute 8-bit × 8-bit, two 4-bit × 8-bit, four 4-bit × 4-bit, sixteen 2-bit × 2-bit, etc., multiplications in one clock cycle. However, Bit Fusion would incur a large area and energy overhead because it requires shift operations and heavy additions for re-configurability. In [31], Mei et al. proposed a 2D symmetric scalable architecture, which used the array multiplier with sum-separate or sum-together modes. These two modes provide the same throughput with different precision (2,4,8,16-bit) at low area and power consumption costs. However, there is a trade-off in this design between input bandwidth and hardware utilization because the input bandwidth in the sum-together case remains the same in all the precision modes at the cost of a partly gated multiplier, even in the case of small precision such as 2-bit or 4-bit.

Similarly, serial approaches also gained attention in re-configurable architectures. In [32], the authors introduced the Unified Neural Processing Unit (UNPU), in which the MAC receives weight inputs with 1-bit iterations, but the input activations are in parallel. Furthermore, right-shifting sequential multipliers have been used, as they require a smaller first stage adder to prevent carry propagation. BitBlade [33] suggests an area-energy efficient accelerator based on Bit Fusion. To reduce the shifter and adder overheads of Bit Fusion, the PE is structured to have a bitwise summation. BitBlade sorts out the 2-bit × 2-bit multiplications that have the same amount of shifts from multiple PEs in [30]. This makes all the BitBricks in each PE have the same number of shifts; hence, only one shifter is needed per PE. Additionally, the adder logic overhead is also reduced. In a nutshell, bit-serial approaches offer benefits in term of interconnections, area or power, but the throughput in such designs is very low.

### 3.3. Logic-Level Accelerators

Since the processing element is the core component and is replicated in systolic array-based accelerators, a few recent works presented the idea of breaking down the MAC logic in pre-processing and post-processing stages to factor out the replicated or redundant hardware [34,35,36]. For example, in [34], the authors suggested the FSA-based tensor processing, in which factorization of booth-encoding and hard multiple (3Y) of multiplicand Y was proposed using radix-8 multiplier. Their designs show noteworthy improvement in large bit-widths (e.g., 16, 32, etc.) or big size SAs, while lesser improvements in small bit-widths (e.g., 8-bit) or small size of SAs. However, the most area and delay complexity in PEs MAC occur due to the carry propagation adder in the feedback loop of the accumulator. This bottleneck was remedied by the authors in [35] that proposed factoring out CPA, thereby enabling the processing in a SA along with its arithmetic elements in an unconventional manner. While this resulted in a reduction in delay and the overall area, the power consumption increased significantly mainly due to an increased sequential area.

To summarize the related work, we have described several recent research works that are relevant but differently aligned as compared to the main theme of our work. While the two varieties of bit-decomposition, i.e., serial and parallel approaches, do offer some advantages, this is not without trading-off other parameters, e.g., area and power cost in parallel architectures versus delay cost in serial architectures. Furthermore, making logic-level modifications including the factoring out of the CPA offers area and latency gains at the cost of higher energy consumption. Various complex power optimization techniques, e.g., using approximate computing, have been identified as well that have been implemented standalone with no specific modifications made, e.g., to the multiplier structure or factoring. Moreover, utilizing approximate computing to achieve power optimization would result in a reduction in the overall design precision as well. These techniques are considerably different than what we are targeting in our proposed design, which are based on modified multiplier decomposition along with combining it with fine-grained power gating using a much simpler route of expressing power intent separately from the logic intent, thereby not having to rely on approximate computing to achieve power efficiency in ML accelerators.

## 4. Proposed Design

### 4.1. General Idea of the Proposed Design

In this paper, a 2D N-SA Gemmini systolic array and its variants are implemented where PEs are connected in a mesh grid. The PEs contain MACs that receive two inputs (*X* and *Y*) to perform multiplication (M=X×Y) and partial accumulation (Z=M+FB) on each clock cycle. FB represents the partially accumulated value in the register, when the accumulation is complete, Z represents the final accumulated product (i.e., dot product). Our assumption in this manuscript is for X and Y to be binary representations while FB and Z can have either binary or alternately redundant binary representations. The main components of a MAC block are the multiplier, the adder and an accumulator register, but critically, N-SA, as discussed previously, has two separate CPAs: one for the multiplier and the other for the accumulator. Nevertheless, a more practical MAC can be found in the literature, termed as a conventional systolic array (C-SA) design that utilizes a CSA to replace the multiplier CPA while the final accumulation is performed using a single CPA [36]. A CSA compresses three inputs into two, termed as redundant binary representation (partial sum and shifted carry), without having to propagate the carry. A single CSA adds two XOR gate delays in its critical path [23,37].

Indeed, this design improves the area and delay in comparison to the N-SA, but the improvement in power is almost negligible, which is very necessary, especially for an edge-based ML workload. Moreover, most of the work in the literature for power improvement mainly deals with sparsity via using data compression and matrix packing algorithms to process the array mapping, thus ensuring a reduction in the quantity of zero values in the mapped arrays [17]. The sparsity can alternately be exploited to power-off the processing element components (e.g., the multiplier and adders) to reduce the power consumption when the input bits are zero.

This can be performed via a non-conventional power gating approach that shuts-off the power to the different modules in the design through the PMU with low power cells, i.e., isolation (ISO) and retention (RET) cells being inserted. These cells are collectively termed as the Power Domain Logic (PDL) in this work. The power gating can be accomplished either by the common power format (CPF) standard endorsed by the Silicon Integration Initiative (Si2) or the essentially equivalent but more popular IEEE standard unified power format (UPF) from Accelera [38,39]. Both offer the additional advantage of expressing power intent separately from the logic-level design description, thus making the process of optimizing power more convenient by making minimal changes to the logic-level description of the design being power gated.

In ML accelerators, we can exploit the PDL in order to power-off the multiplier when either *X* or *Y* or both X,Y are zero. Therefore, initially the PDL was applied to the C-SA variant of Gemmini that improved the power efficiency quite substantially, though with a noticeable increase in the area, but due to the conventional nature of the C-SA structure and additional isolation/retention cells, the multiplier delay became very high, making it a dominant factor in the C-SA critical path delay.

In the systolic arrays with a power-intent description, we can actually benefit from the divide and conquer rule by dividing the large multipliers of the PEs into small sub-multipliers. It can help in two ways: (1) The power of sub-multipliers can be shut-off through PDL; (2) however, PDL shall always dominate the design critical path due to the additional delay incurred by the PDL cells. Since the PDL is applied at either the 8-bit multiplier (in C-SA) instance or the 4-bit sub-multiplier instance, so the design critical path would either be through the 8-bit multiplier or the 4-bit sub-multiplier. Thus, it is obvious that the four 4-bit sub-multipliers in parallel would give an advantage over a single 8-bit multiplier. To divide the large multiplier, bit-decomposition can be performed, which is widely used in re-configurable systolic array architectures. However, in conventional bit-decomposition-based multiplication, each multiplier contains a small CPA, which is still a bottleneck in the overall performance improvement. Therefore in this work, the bit-decomposed multiplication was restructured by replacing each sub-multiplier CPA with a CSA. We can thus term the proposed design with power-intent description and structurally redesigned decomposed multiplier as a power-intent systolic array (PI-SA) design. However, power saving through UPF comes at the cost of additional PDL circuitry, thereby resulting in an additional area cost that in turn is replicated in all the PEs of the SA, which can be offset by CPA factorization at the systolic array level [35].

**Figure 3:**
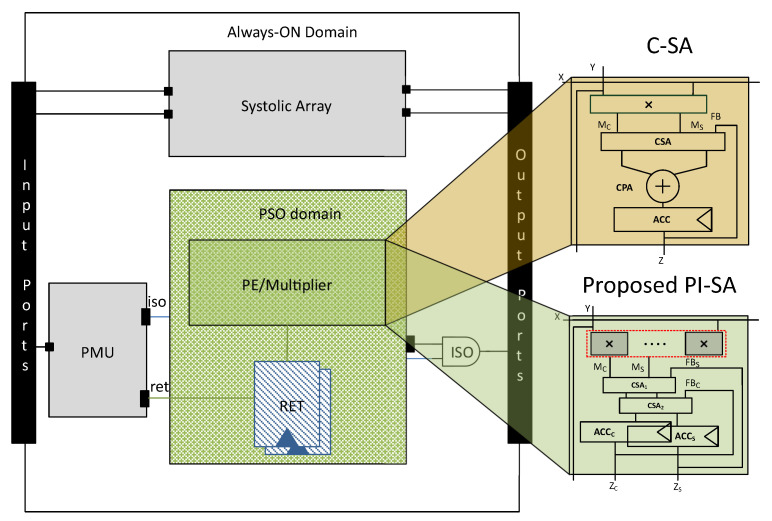
Generalized view of the PMU with C-SA and the proposed design.

We have depicted a generalized view of the C-SA and the proposed PI-SA design (for simplicity, a single accumulator register is used in both SAs) along with the PMU and the relevant power domain representations in Figure 3.

### 4.2. Multiplier Decomposition

Bit Fusion [30] introduced the BitBricks concept through bit-decomposition of a large multiplier to accomplish re-configurable systolic array accelerators. BitBricks performs 2-bit × 2-bit multiplication and together with the bit decomposition concept, any variable length multiplication can be achieved.

For example, for an 8-bit × 8-bit bit-decomposition multiplication, the multiplier $X=x_{7}x_{6}\cdots x_{1}x_{0}$ and multiplicand $Y=y_{7}y_{6}\cdots y_{1}y_{0}$ can be divided into four 4-bit groups, as shown in (3).

(3) $$\begin{array}{cc} X_{LSB} & =x_{3}x_{2}x_{1}x_{0},\;X_{MSB}=x_{7}x_{6}x_{5}x_{4}\\ Y_{LSB} & =y_{3}y_{2}y_{1}y_{0},\;Y_{MSB}=y_{7}y_{6}y_{5}y_{4} \end{array}$$

**Figure 4:**
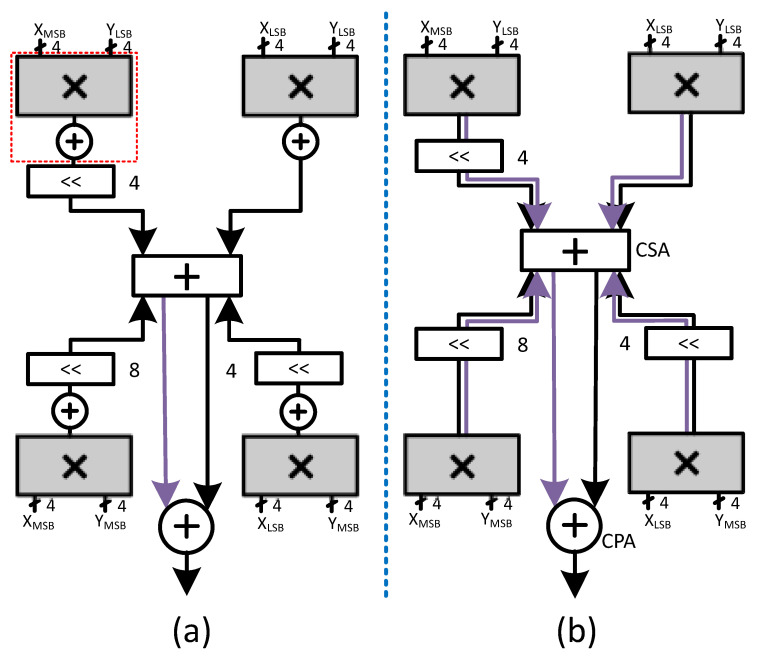
Bit-decomposed multiplier. (**a**) Conventional. (**b**) Redesigned.

Using these four groups of 4-bits each, the decomposed multiplication can be performed in the following steps: (1) decomposed products generation, which can be accomplished through four 4-bit multipliers such as radix-2 or radix-4; (2) three decomposed products shifting through left shifters for correct alignment; (3) decomposed products reduction tree (e.g, Wallace Tree); and (4) the final addition of the last two partial products through carry propagation adder to obtain the product, as shown in Figure 4a. We summarize the whole process generically in (4).

(4) $$\begin{array}{cc} M_{00} & =X_{LSB}\times Y_{LSB}\\ M_{01} & =X_{MSB}\times Y_{LSB}\\ M_{10} & =X_{LSB}\times Y_{MSB}\\ M_{11} & =X_{MSB}\times Y_{MSB}\\ M & =\left(2^{2n}\times M_{11}\right)+\left(2^{n}\times M_{10}\right)\\ & +\left(2^{n}\times M_{01}\right)+\left(2^{0}\times M_{00}\right) \end{array}$$

Here, *n* represents the bit-decomposition bit-width, or the number of bits in each group. Since the bit-decomposition requires a group of four multipliers, if we use the conventional radix-2 or radix-4 multiplier for each multiplication then, we would require four small 8-bit CPAs too inside each multiplier. Therefore, in a conventional bit-decomposed multiplication, (effectively BitBrick in [30] or Radix-4), the carry propagation adder is replicated in each small multiplier thus, adding an additional area, delay and power cost. We can replace these CPAs inside each small multiplier with CSAs and put a single CPA at the output to obtain the final product. Indeed, this will make the interconnections and shifters logic cost double, but at the same time, we can get rid of the area, delay and power cost of using four CPAs. Figure 4b shows the modified version of our bit-decomposed multiplier.

### 4.3. C-SA

**Figure 5:**
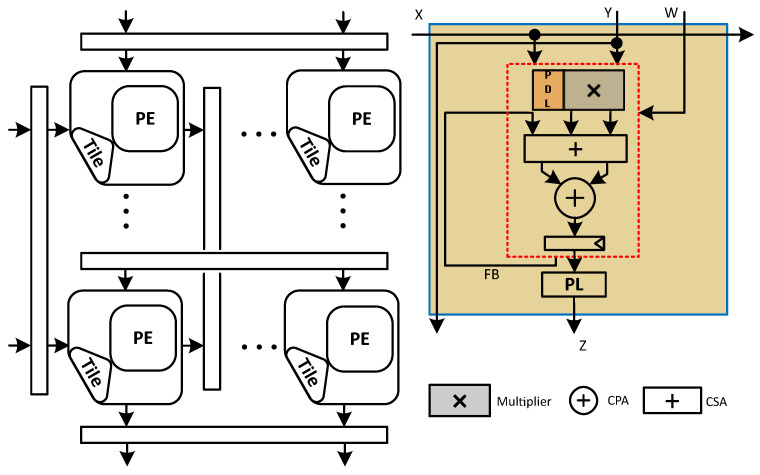
Broad micro-architecture of the C-SA design.

A micro-architecture of an 8×8 power-intent Gemmini variant conventional systolic array (C-SA) is shown in Figure 5, which performs multiplication on 8-bit signed inputs and accumulation with 32 bits.

Contrary to the N-SA, in C-SA MAC, we merged the multiplier (the structure-level 2’s complement signed radix-4 multiplier with complete sign extension is used) with the accumulator: Instead of using a CPA for the final addition of the two partial products, we used CSA with the third value, i.e., FB, stored in the accumulator register. As a result, we used just a single CPA to determine the final value, as explained in [36].

Initially, we applied the PSO logic on each PE multiplier of the C-SA. Lets say, $X_{row}\;\mathrm{and}\;Y_{col}$ are row×col systolic array inputs, where $X_{row}$ are given at the left edge (in rows) and $Y_{col}$ at the top (in columns). If $X_{row}$ is zero, then with the help of the retention cells, the previous outputs of the multipliers in the whole row of PEs, i.e., $M_{row}$ is retained and similarly isolation cells are also placed in a row-wise fashion. Similarly, if $Y_{col}=0$, then the same logic is applied on the whole PE multipliers column-wise. We have represented it with a PDL block and highlighted it with orange color along with the multiplier in Figure 5. Nevertheless, when $X_{row}\;\mathrm{and}\;Y_{col}$ are both zero then, the whole row and whole column PE multipliers go into the PSO state. The algorithmic description of this whole procedure is shown in Algorithm 2, which shall in principle result in the pso_enable signal and subsequently the PDL according to the logic presented in the Algorithm 1.
**Algorithm 2:** Generalized pso_enable generation algorithm**Input**: Systolic Array Input signals $X_{row}$, $Y_{col}$;**Output**: PSO enable signal resulting in multiplier shutoff;**if** *$(X_{row}==0\;$ &&$\;Y_{col}==0$*) **then**(  $M_{row}\;⟹$ off;  $M_{col}\;⟹$ off;**end**(**else if** $(X_{row}==0$) **then**(  $M_{row}\;⟹$ off;**end**(**else if** $(Y_{col}==0$) **then**(  $M_{col}\;⟹$ off;**end**(

In this way, the PDL consisting of state retention and isolation cells inserted through UPF saves power significantly. However, this improvement in C-SA is achieved at the cost of an increased delay of the multiplier due to the PDL, which makes it the critical path of the systolic array. As we used an 8-bit Radix-4 multiplier, so as per the equation $\left\lceil \frac{\left(N+1\right)}{r}\right\rceil$ in Section 2.1, the number of the total partial product equals: $\lceil \frac{\left(8+1\right)}{2}\rceil =5$. Thus, the multiplier delay can be reduced by decreasing the number of partial products, as explained in the subsequent section.

### 4.4. Proposed PI-SA Design

**Figure 6:**
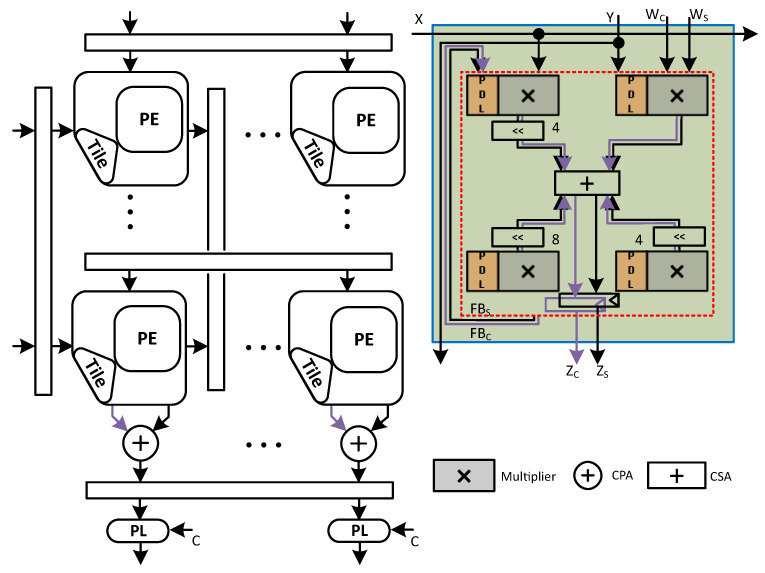
Broad micro-architecture of the proposed PI-SA design.

The micro-architecture of the proposed PI-SA design in Gemmini configuration with an output stationary dataflow is shown in Figure 6. This design also utilizes an 8-bit multiplier and a 32-bit accumulator. The proposed design divides the 8-bit multiplier into four sub-multipliers (4-bit each) inside the PE MAC, using a bit-decomposition technique to reduce the partial products and a single CPA for the final product as explained in Section 4.2 and shown in Figure 4b.

Now that we have four 4-bit sub-multipliers, we can reduce the five partial products into $\lceil \frac{\left(4+1\right)}{2}\rceil =3$ partial products using radix-4 multipliers. This would then require the usage of only one CSA (3:2 compressor) for the last two partial products. On the contrary, in the case of a single 8-bit multiplier, we shall require three CSAs to reduce the five partial products to the final two partial products. In this way, the number of XOR gates in the reduction tree with the 4-bit sub-multiplier is reduced from six to two. Therefore, even though the PDL logic is added to each sub-multiplier (one per PD), with four of them functioning in parallel with no CPA, this sub-multiplier critical path would reduce considerably in comparison to a single multiplier (per PD) that constituted the critical path in the C-SA case. The addition of the final eight partial products (two from each sub-multiplier) thereafter is accomplished through CSAs placed outside the sub-multipliers.

Additionally, this bit-decomposition will increase the number of multiplier shut-off instances in general as compared to the C-SA design described in Section 4.3. This is due to an increase in the PEs/multipliers/PDs in the proposed SA design as compared to the C-SA design as explained in the succeeding Section 4.5. This increase in the multiplier shut-off instances can be demonstrated with the help of an example where only the $X_{row}$ input of the SA equals zero in the case of C-SA. According to Algorithm 2, this shall result in the shutting-off of the row multipliers $M_{row}$. The consequence of the $X_{row}$ input equaling zero would, however, be that the values of both the $X_{LSB}$ and $X_{MSB}$ inputs (ref. to Equation (3)) to the bit-decomposed multipliers $M_{00},M_{01},M_{10},M_{11}$ (ref. to Equation (4)) would be equal to zero. This shall, hence, result in shutting-off of four instances of sub-multipliers in PI-SA design for every single multiplier instance in the C-SA design. Additionally, for a C-SA multiplier to be shut-off, the whole 8-bit PE input needs to be zero, while in the case of PI-SA, two of the four sub-multipliers corresponding to each C-SA multiplier would be shut-off even with either the LSB or the MSB of any specific PE input equaling zero as indicated by Equation (4).

Furthermore, because we divided a single 8-bit multiplier with 16-bit output into four 4-bit multipliers (working in parallel), each producing two 8-bit outputs in redundant binary representation (partial sum and shifted carry), the interconnections have been quadrupled (8×4×2=64-bit instead of 16-bit) in comparison to a single multiplier. As a result, the PDL was increased along with the doubling of the shifters, thus, resulting in a degradation in the total area of the systolic array.

Moreover, since the sequential cells consume more power than the combinational cells, it led to a slight degradation in power improvement, but this can be accommodated since we already save enough power by increasing the opportunity of power gating the sub-multipliers as explained before. Lastly, as the effect of CPA factorization at SA level will also be added, so this new design provides handsome improvement in the overall area too, as explained in detail in the subsequent section.

### 4.5. Cost of Low Power Logic in the Systolic Arrays

The insertion of low power cells results in power optimization at the cost of additional low power logic in the form of isolation and state retention cells inserted in the switchable power domain. We have performed optimizations on the C-SA and the proposed PI-SA varieties of the test cases. Let 2P be the multiplier output register bitwidth per PE/PD in C-SA and Q be the number of power domains in C-SA. The multiplier output register bitwidth per PE in the PI-SA design hence is P while, due to the multiplier decomposition, the number of power domains in this case is 4Q. The number of low power isolation and retention cells (${\mathrm{LP}}_{\mathrm{FF}/\mathrm{ISO}}$) in each case can be represented mathematically by (5). Table 1 shows the number of power domains as well as the number of low power cells for each design test case in accordance with (5).

(5) $$\begin{array}{cc} {\mathrm{LP}}_{\mathrm{FF}/\mathrm{ISO}} & =\left\{\begin{array}{cc} 2\mathrm{PQ}, & C\text{-}\mathrm{SA}\\ 4\mathrm{PQ}, & \mathrm{PI}\text{-}\mathrm{SA} \end{array}\right. \end{array}$$

## 5. Evaluation

This section presents the evaluation methodology in addition to the experimental results indicating the execution of the various designs with respect to the performance objectives. We begin by experimenting with the systolic array utilizing the 32-bit-width accumulator and considering the N-SA design utilizing the baseline Gemmini-based systolic array. This is then followed by two designs that are optimized in various aspects. While the C-SA design is optimized only by incorporating the power intent and the required logic-level changes to describe the intent, the PI-SA design involves our proposed optimizations both at the logic-level (primarily to restructure the decomposition of the 8-bit multiplier into multiple 4-bit sub-multipliers by replacing each sub-multiplier CPA by CSA) and at the power level (to introduce power intent). We then also demonstrate the impact of our optimizations for varying precision by performing experiments with systolic arrays involving 16-bit- and 64-bit-wide accumulators.

### 5.1. Evaluation Methodology

All the designs have been synthesized using SAED 32nm process technology and the design compiler by Synopsys. RTL and netlist verification have been carried out using VCS simulator, which has been used in combination with prime time tool for obtaining the switching information using SAIF file. Random synthetic vectors have been used for the design verification and SAIF file generation while Prime Power tool has been used for power analysis in the *averaged* power mode. Null values are randomly spread in the input vectors to mimic the sparsity portrayed by numerous ML applications and these null values would result in the power gating of the switchable power domain. A Linux machine with 128 GB of memory has been used for performing all the experiments. The baseline design is the naive systolic array, while we have also power gated the conventional systolic array presented in [36] and then compared the results of both these designs with our proposed PI-SA design.

### 5.2. 32-Bit Wide Accumulator Systolic Arrays

A comparison of the various SA test cases with the 32-bit-wide accumulator based on the five performance parameters is presented in Table 2. The delay is increased in the case of C-SA due to the delay incurred by the low power cells even with the multiplier CPA of the N-SA replaced with CSA in this design. This also results in area reduction even though low power cells are inserted. There is a further increase in area in the PI-SA design as compared to the C-SA due to the additional sequential logic resulting from the extra retention cells. The area is still, however, lower as compared to N-SA due to the CPA replacement from each sub-multiplier as well as the PE datapath. The power savings in the cases with UPF are evident from Table 2, but the power improvement of the PI-SA case in comparison to N-SA is compromised slightly with regards to the comparison of C-SA with N-SA due to the additional PDL incurred in the PI-SA design. The combined effects on delay and power (PDP) as well as the delay and area (ADP), however, by the optimizations in our proposed PI-SA case, outweigh those of the other two cases significantly in general with the ADP of the PI-SA design almost the same as that of the best case ADP, i.e., of the N-SA design.

**Figure 7:**
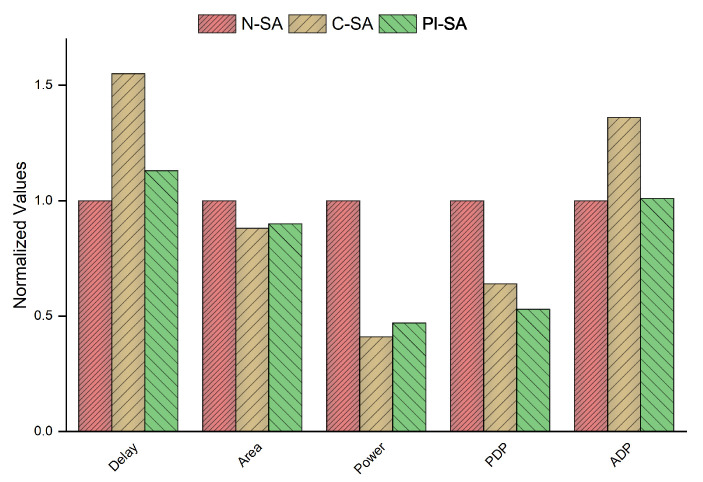
Comparison between the normalized values of the performance parameters for 32-bit wide accumulator.

The results in normalized form are further compared graphically in Figure 7 to clearly depict the impact on various performance parameters in percentages for all the designs under test. The lower the value of a parameter for a specific design test case, the better shall the design be considered based on that design parameter.

The N-SA has the least delay primarily because of the low power logic inserted in the other design test cases that incurs additional delay in the design. Among the power gated designs, the PI-SA case incurs the largest number of low power cells, but with a smaller reduction tree due to the multiplier decomposition. The delay cost of the PI-SA design as compared to the N-SA design, i.e., around 13%, is smaller as compared to that of the C-SA design in comparison to the N-SA, i.e., greater than 55%. The area cost of the N-SA is the highest followed by the C-SA (area saving of approximately 13% compared to the N-SA) and the PI-SA design (area saving of about 11% compared to the N-SA). The area improvement of the PI-SA case is, however, slightly compromised as compared to the area improvement of the C-SA case due to the additional sequential logic resulting from the increased retention cells and output propagation in redundant binary representation in the PI-SA design in addition to the larger register size in the accumulator of the PI-SA design. As far as the power efficiency is concerned, the power gated designs exhibit higher performance efficiency as clear from the Figure 7. The power savings in the PI-SA case (53% saving compared to the N-SA) are again slightly lower as compared to the C-SA (59% saving compared to the N-SA) case mainly due to the additional retention logic and the larger accumulator registers sizes, thus resulting in a larger sequential area and hence higher power consumption comparatively. The proposed PI-SA design, however, performs the best in terms of the PDP and ADP among all the test cases. It exhibits a PDP improvement of around 47% as compared to the PDP improvement of 36% of the C-SA when compared with N-SA and an ADP improvement of around 35% as compared to the C-SA relevant to the N-SA design.

### 5.3. Different Bitwidth Accumulator Systolic Arrays

This section consists of the results for varying accumulator bitwidths for all the designs under test. We have performed the comparison of our proposed PI-SA design with the other two designs in terms of delay, area, power, PDP and ADP considering 16-bit, 32-bit and 64-bit wide accumulators. The purpose of this comparison is to evaluate the impact of our proposed optimizations for varying precision of SAs so as to demonstrate that different SA accumulator sizes will yield different results in terms of performance comparison considering the aforementioned performance objectives.

**Figure 8:**
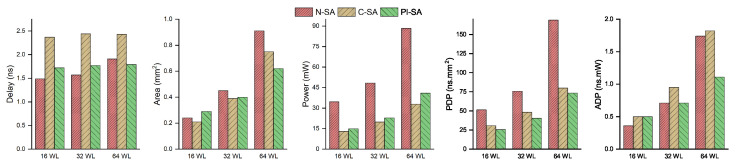
Performance comparison of proposed PI-SA, C-SA and N-SA.

**Figure 9:**
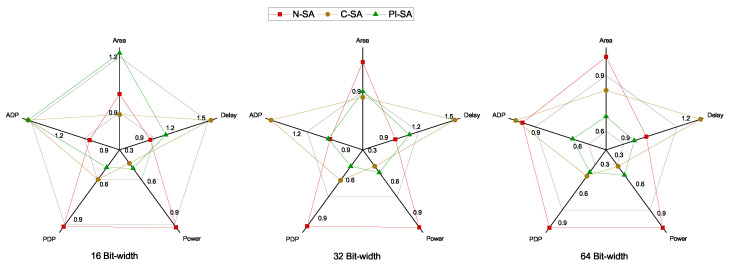
Comparison between the normalized values of the performance parameters.

The comparative results in terms of the absolute values of the various performance parameters are given in Table 3. The same values are depicted graphically in Figure 8 as well. The comparative assessment of the various designs in terms of the performance objectives is portrayed graphically in Figure 9 depicting normalized performance parameters wherein, a lower value on any parameter axis would indicate a better performance of the design with respect to that parameter. The proposed PI-SA design in terms of the delay performance in the case of higher bitwidth SAs, i.e., 64-bit SA, is able to beat the baseline N-SA by achieving an improvement of around 6%, as is clear from the Figure 9. This is because, in the N-SA, since the multiplier is cascaded with the CPA (so basically there are two CPAs, one for last two partial products addition inside multiplier and one for the processing element MAC), so the critical path is occurring in between the pipeline register and the accumulation register, which contains a huge two CPA carry propagation delay. However, in the proposed design case, firstly, we decomposed the large multiplier into smaller sub-multipliers; secondly, we also took all the CPAs out from the processing elements. Moreover, we also took the large reduction tree outside the multiplier (inside the multiplier, the reduction tree is small), so in this case, the critical path is occurring in the power optimization logic rather than the multiplier or the CPA, thus causing no carry propagation delay with smaller accumulator bit-widths. However, in the 64-bit-width case, because of large carry propagation in both the designs, the critical path is on the MAC in N-SA, while in our proposed design, the CPA is outside the processing element, thus leading to its comparatively superior delay performance.

The area performance of PI-SA design for the 64-bit case depicts an improvement of around 32% as compared to the N-SA design owing to the drastic improvement in the combinational area in the case of higher precision as compared to the 32-bit accumulator design. Moreover, the 64-bit PI-SA also portrays a lower area than the C-SA case. As stated before, the area in the case of PI-SA is improved mainly due to the reduced combinational area since the sub-multiplier CPAs are factored out. The C-SA case, however, consists of a CPA for the final accumulation. This results in the area cost of PI-SA catching up with C-SA as the accumulator width increases and even beats the C-SA when the accumulator width reaches 64. As far as the power performance is concerned for the 64-bit accumulator case, the power efficiency of our proposed design in comparison to the baseline N-SA is consistent with that of the 32-bit accumulator case, i.e., around 53–54%. The cumulative PDP and ADP comparison from Figure 9 clearly indicates an improvement of around 56–57% and 36% for our proposed PI-SA design in comparison to the N-SA design, respectively, for the 64-bit accumulator owing to better overall performance of the proposed design with respect to all the other individual performance parameters.

## 6. Conclusions

This manuscript presents our efforts in optimizing ML acceleration by utilizing the Gemmini SA micro-architecture as the baseline. In particular, our optimizations are based on introducing power-intent in the C-SA variant of the baseline design by using the UPF industrial standard, thus resulting in around 59% saving of the design power consumption. This, however, resulted in an increased delay by 55% in the C-SA design due to the additional power logic, which was further complimented by a single 8-bit multiplier dominating the design critical path. This was thereby remedied by proposing a novel PI-SA design with further micro-architectural modifications to divide the large 8-bit multiplier into four smaller 4-bit sub-multipliers, thus resulting in a smaller number of partial products and the corresponding delay reduction of more than 40% as compared to the C-SA. This was performed at a small area improvement cost of around 2% and power improvement cost of around 6% due to the additional sequential logic resulting from the doubling of the accumulator registers of the proposed PI-SA design in addition to the increased retention logic emanating from the increased power domains in the proposed design. The overall improvement in PDP and ADP, however, was quite profound in the proposed design as compared to the C-SA design when observed with respect to the N-SA design.

However, our further analysis considering higher precision with a wider 64-bit accumulator demonstrated a better performance of our proposed design with respect to all the performance parameters ranging from a minimum of 6% delay reduction to a maximum of around 57% performance improvement in the PDP performance as compared to the N-SA design. For future research, we intend to compliment this work with further macro-architectural power optimizations, e.g., to reduce frequent memory-accesses that are a major hurdle in the development of power-efficient edge AI devices.

## Figures and Tables

**Table 1:** Low power cells in various design test cases.

Design	# Power Domains	# ISO Cells	# RET Cells
C-SA	64	2048	2048
PI-SA	256	4096	4096

**Table 2:** Performance comparison of an 8×8 SA with 32-bit accumulator.

Design	Delay (ns)	Area (mm^2^)	Power (mW)	PDP (ns·mW)	ADP (ns·mm^2^)
N-SA	1.57	0.45	48.30	75.83	0.70
C-SA	2.44	0.39	19.80	48.31	0.95
PI-SA	1.77	0.40	22.8	40.35	0.71

**Table 3:** Implementation results of the proposed PI-SA, N-SA and C-SA with different accumulator bit-widths N = 16, 32 and 64.

Design	Acc (N)	Delay (ns)	Area (mm^2^)	Power (mW)	PDP (ns·mW)	ADP (ns·mm^2^)
N-SA		1.49	0.24	34.6	51.55	0.35
C-SA	16	2.37	0.21	13.0	30.81	0.50
PI-SA		1.72	0.29	14.90	25.62	0.49
N-SA		1.57	0.45	48.30	75.83	0.70
C-SA	32	2.44	0.39	19.80	48.31	0.95
PI-SA		1.77	0.40	22.80	40.35	0.71
N-SA		1.91	0.91	88.40	168.84	1.74
C-SA	64	2.43	0.75	32.90	79.95	1.83
PI-SA		1.79	0.62	41.0	73.39	1.11
